# Twins Born after an Interval of Two Months

**Published:** 1848

**Authors:** 


### Article VIII.—*Twins born after an interval of two months: they live.* Translated from the Journ. des Con. Medico-Chirurg.

Dr. Wilberg reports a young lady, married ten months, who after eight months pregnancy was delivered of an infant on the 24th of March, which lived, but had not attained its full uterine development. The accouchment was laborious; the placenta was discharged spontaneously but the mother had no nourishment for the child. She thought she felt movements of another child, and the abdomen increased in size. The 20th of May she was again taken in labor, and gave birth to a child more robust and heavier than the first one. Three days after this she was enabled to nurse both her children.—*South. Med. Journ.*

